# Approach to record linkage of primary care data from Clinical Practice Research Datalink to other health-related patient data: overview and implications

**DOI:** 10.1007/s10654-018-0442-4

**Published:** 2018-09-15

**Authors:** Shivani Padmanabhan, Lucy Carty, Ellen Cameron, Rebecca E. Ghosh, Rachael Williams, Helen Strongman

**Affiliations:** 1 Clinical Practice Research Datalink (CPRD), MHRA, 10 South Colonnade, Canary Wharf, London, E14 4PU UK; 2 NHS Digital, 1 Trevelyan Square, Boar Lane, Leeds, LS1 6AE UK

**Keywords:** Electronic health records, Record linkage, Deterministic linkage, Primary care data, Clinical Practice Research Datalink

## Abstract

Record linkage is increasingly used to expand the information available for public health research. An understanding of record linkage methods and the relevant strengths and limitations is important for robust analysis and interpretation of linked data. Here, we describe the approach used by Clinical Practice Research Datalink (CPRD) to link primary care data to other patient level datasets, and the potential implications of this approach for CPRD data analysis. General practice electronic health record software providers separately submit de-identified data to CPRD and patient identifiers to NHS Digital, excluding patients who have opted-out from contributing data. Data custodians for external datasets also send patient identifiers to NHS Digital. NHS Digital uses identifiers to link the datasets using an 8-stage deterministic methodology. CPRD subsequently receives a de-identified linked cohort file and provides researchers with anonymised linked data and metadata detailing the linkage process. This methodology has been used to generate routine primary care linked datasets, including data from Hospital Episode Statistics, Office for National Statistics and National Cancer Registration and Analysis Service. 10.6 million (M) patients from 411 English general practices were included in record linkage in June 2018. 9.1M (86%) patients were of research quality, of which 8.0M (88%) had a valid NHS number and were eligible for linkage in the CPRD standard linked dataset release. Linking CPRD data to other sources improves the range and validity of research studies. This manuscript, together with metadata generated on match strength and linkage eligibility, can be used to inform study design and explore potential linkage-related selection and misclassification biases.

## Introduction

The widespread digitisation of health records in the UK and worldwide over the past two decades has created an exponential growth in the secondary use of routinely-collected healthcare data for research [1]. Clinical Practice Research Datalink (CPRD) is a UK Government research service jointly supported by the Medicines and Healthcare products Regulatory Agency (MHRA) and the National Institute for Health Research (NIHR) to promote healthcare research and drive innovation through use of UK patient electronic health records (EHR). CPRD provides anonymised UK EHRs to researchers within academic, regulatory, and pharmaceutical organisations worldwide to support observational public health research [2].

Record linkage is increasingly used to combine information from different sources and generate rich, comprehensive data for research, policy and health services planning [3–6]. Whilst internationally there are many examples [7–12] of record linkages, CPRD was the first to provide routine record linkages between primary care data and a range of health-related patient datasets within England (Box 1). CPRD provides access to these data following approval of a research protocol in accordance with data governance procedures and research ethics. Currently more than two-thirds of research protocols submitted to CPRD request the use of linked data. Record linkage increases the information available on patient care, diseases and conditions, expanding the opportunity for research and strengthening the knowledge gained from primary care [13–17]. However, the potential for false or missed matches in the linkage process, and in subsequent analyses, can introduce selection and misclassification biases in research studies [5, 18, 19]. Recent guidelines for the reporting of observational studies indicate that sharing information on the record linkage process may improve interpretation of study findings and maintain the validity of linked data as a valuable research resource [3–5, 20]. The 2017 GUidance for Information about Linking Datasets (GUILD) publication outlined suggestions for both data linkage service providers and data users on the reporting of linkage methodology and analysis of linked data [3]. In line with the GUILD suggestions, and reporting guidelines for observational research, this paper describes the approach to record linkage used by CPRD and NHS Digital, a statutory body in England, permitted to receive identifiable patient data for linkage.

**Box 1:** CPRD routine linkages

Hospital Episode Statistics Admitted Patient Care (HES APC)
Hospital Episode Statistics Outpatient (HES OP)
Hospital Episode Statistics Accident and Emergency (HES A&E)
Hospital Episode Statistics Diagnostic Imaging Dataset (HES DID)
Office of National Statistics (ONS) Death Registration
National Cancer Registration and Analysis Service (NCRAS) data from Public Health England (PHE) including:
Cancer registration data
Cancer Patient Experience Survey (CPES) data
Systemic Anti-Cancer Treatment (SACT) data
National Radiotherapy Dataset (RTDS)
Mental Health Dataset (MHDS) data
Measures of relative deprivation and rural urban classification at Lower Layer Super Output Area (LSOA) level for practices and patients

This paper describes the record linkage methodology, in order to improve understanding among researchers and encourage the incorporation of linkage methodology into the design, analysis and reporting of epidemiological studies.

## Methods

### Data governance and ethics

CPRD operates a general practice ‘opt-in’ and patient ‘opt-out’ system. GP practices choose to contribute de-identified patient data to CPRD for all patients, with the exception of those who have opted-out from the sharing of their patient record with CPRD or NHS Digital. GP practices must also give consent for their patient data to be linked.

CPRD has broad annual research ethics approval from the UK’s Health Research Authority (HRA) Research Ethics Committee (REC) to receive and supply patient data for purely observational public health research using the primary care data and established data linkages. CPRD also has Section 251 regulatory approval annually renewed from the HRA to supply anonymised linked data from English general practices for public health research. Appropriate regulatory approval must also be obtained by each data custodian for their dataset to be linked to CPRD primary care data.

Data linkage is enabled by NHS Digital, the statutory body in England legally permitted to receive patient identifiable data. CPRD and NHS Digital have a data sharing agreement in place governing the linkage process.

Observational research undertaken using CPRD data must be for public health purposes and approved by an Independent Scientific Advisory Committee (ISAC). Following ISAC approval, contractual controls ensure researchers adhere to robust terms and conditions governing data use.

### Data flow

CPRD has established a data linkage programme that routinely links primary care data to other patient-level health data from data custodians NHS Digital and Public Health England (PHE).

Data linkage is undertaken by NHS Digital, known in law as the Health and Social Care Information Centre (HSCIC), the national provider of information, data and IT systems within health and social care in England, and the statutory body in England legally permitted to receive identifiable patient data.

#### Primary care data flow

**Fig. 1:**
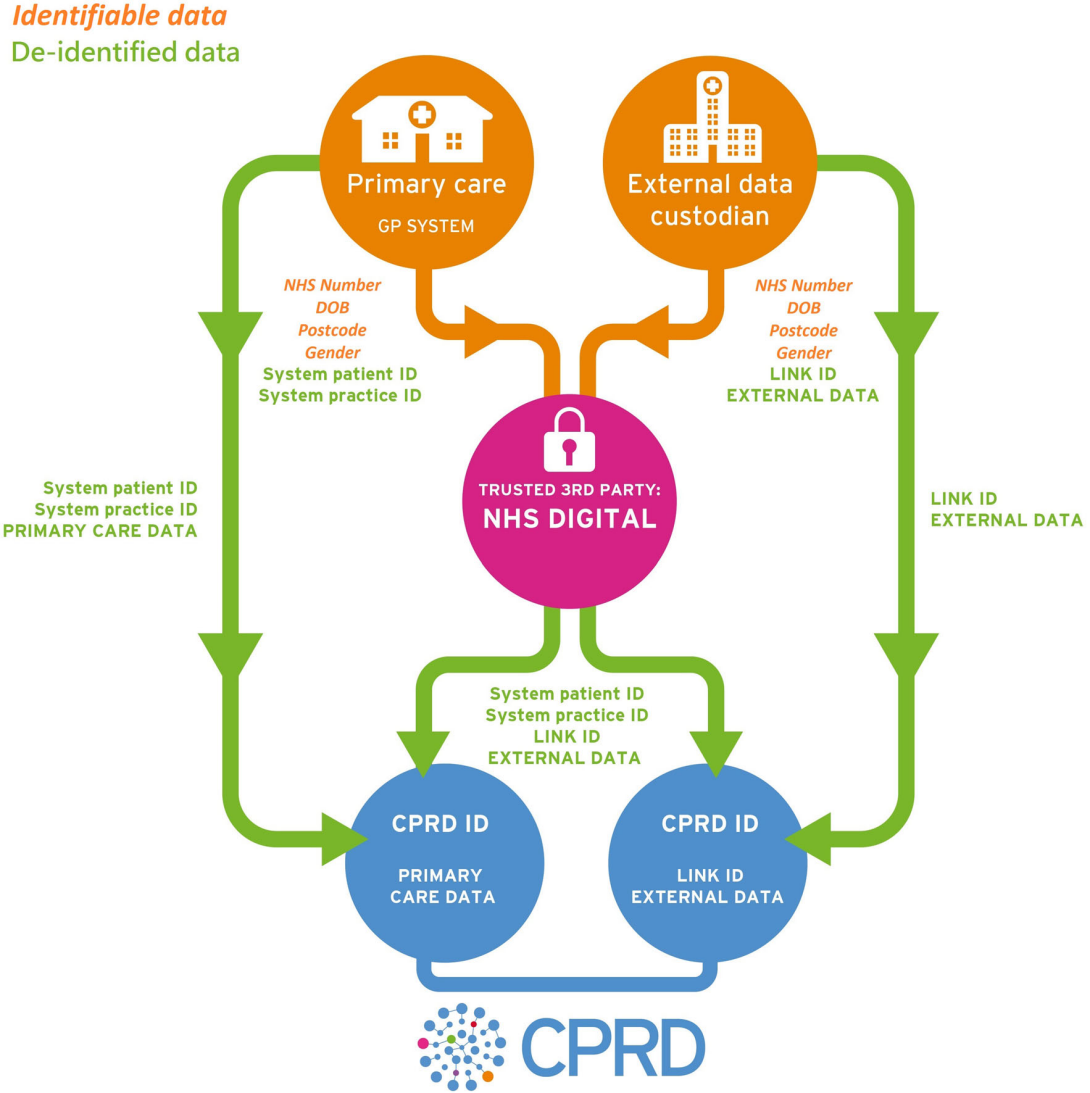
Primary care and linked data flow. De-identified linked data can either flow from external data custodians to NHS Digital and subsequently to CPRD, or directly from external data custodians to CPRD

Primary care data are submitted to CPRD via general practice electronic software suppliers acting as data processors. Data are submitted on a regular basis from practices that have agreed to contribute data (Fig. 1). Personal identifiers including name, full date of birth, postcode and National Health Service (NHS) number are removed at source by the system provider and replaced by pseudonymised system patient and practice identifiers prior to transfer of data to CPRD. Data from patients who have registered to opt-out at a contributing practice are not provided to CPRD.

For practices that have consented to participate in record linkage, general practice software suppliers submit personal identifiers (NHS number, gender, date of birth and postcode) to NHS Digital, alongside system patient and practice identifiers (Box 2). Flags are generated by NHS Digital to indicate the validity of the NHS number, gender and postcode fields, and data are cleaned to remove duplicate records and to validate the removal of patients who have opted out of data sharing. Data are then merged with the previous data submission to retain the latest available information for patients from practices that are no longer contributing data to CPRD, and to ascertain patients that are ‘new’ to linkage since the previous submission. The cleaned and merged data constitute a CPRD cohort file, ready for linkage to other patient-level health data.

**Box 2:** Data submitted to NHS Digital. *Italicized text* indicates personal identifier used for linkage

General practice system providers	External data custodians
System patient identifier	Patient linkage identifier
System practice identifier	*NHS number*
*NHS number*	*Date of birth*
*Date of birth*	*Gender*
*Gender*	*Current Postcode*
*Current Postcode*	

#### Secondary care and other health related data flow

External data custodians submit personal identifiers (NHS number, gender, date of birth and postcode) to NHS Digital, alongside a pseudonymised patient record identifier, known as a link ID, for all patients in their dataset (Fig. 1; Box 2). NHS Digital matches identifiers submitted by external data custodians to the primary care identifiers in the CPRD cohort file, generating a linker file. The linker file contains a pair of pseudonymised identifiers (GP system patient and practice ID, external dataset link ID) for each linked patient that can be used to merge the primary care dataset with the external dataset.

Following linkage, de-identified linked data can flow via two distinct routes (Fig. 1). In the first instance, de-identified linked data flows directly from NHS Digital to CPRD. The linked dataset contains full back data for new patients and incremental data for existing patients. Patients who have opted in following a prior opt-out are treated as new patients and full back data are included. In the second instance, NHS Digital supplies CPRD with linker files only and CPRD requests relevant data for selected link IDs or specified patient cohorts from external data custodians, based on researcher needs.

### Linkage strategy and metadata

The goal of record linkage is to determine whether records link to the same or different units of observation, in this case an individual patient, using identifiers that are common among datasets. Identifiers may be unique (e.g. NHS number) or partial, i.e. partially identifying characteristics that may be shared by more than one individual or may change over time, such as postcode, or ‘incomplete’ identifiers such as year of birth or first two digits of a postcode. Accurate record linkage is dependent on the discriminatory power of available identifiers (Box 2), the overall quality of the datasets, and the design of the deterministic or probabilistic linkage strategy [19]. Deterministic linkage strategies use rules based on agreement between variables. Probabilistic strategies calculate scores for each variable based on the probability of observing an agreement between variables. Score thresholds are usually set to classify matches [21].

Identifiers in the CPRD cohort file are matched with identifiers from external data custodians through an iterative deterministic method comprising a series of eight progressively less restrictive steps generated from combinations of NHS number, date of birth, postcode and gender. The step at which a primary care record matches an external record is recorded as the match rank. Records matched at a given step are not available for matching in subsequent steps.

NHS Digital supply CPRD with metadata generated during the linkage process relating to eligibility and match quality (Table 1). The CPRD cohort file contains all patients participating in record linkage and includes the following fields to determine eligibility for linkage based on the availability of identifiers:

**Table 1:** Deterministic linkage steps

Step (match rank)	Match required
1	Exact NHS number, gender, DOB and postcode
2	Exact NHS number, gender and DOB
3	Exact NHS number, gender, postcode and partial DOB
4	Exact NHS number, gender and partial DOB
5	Exact NHS number and postcode
6	Exact gender, DOB and postcode (NHS number must not contradict the match, DOB must not be 1st of January and postcode must not be on the communal establishment list^a^)
7	Exact gender, DOB and postcode (NHS number must not contradict the match and DOB must not be 1st of January)
8	Exact NHS number

^a^Communal establishments include: hospitals, care homes, prisons, defence bases, boarding schools and student halls of residence

- *System patient ID and practice ID* pseudonymised patient identifiers that allow integration with the CPRD primary care record
- *NHS flag* indicates whether the patient had a valid NHS number in the primary care record
- *DOB flag* indicates whether the patient had a valid date of birth in the primary care record
- *Postcode flag* indicates whether the patient had a correctly formatted postcode in the primary care record
- *Link date* date when personal identifiers required for linkage were sent by the primary care system provider to NHS Digital

The linker file provided with each linkage contains only patients where a match has been identified and includes metadata on the quality of each matched record as defined in Table 1. Linker files contain the following fields:

- *System patient ID and practice ID* pseudonymised patient identifiers that allow integration with the CPRD primary care record
- *Link ID* pseudonymised patient record identifiers that allow integration with the external dataset being linked to
- *match_rank* indicates the quality of matching between CPRD and the external dataset and corresponds to the step at which the match was established. This is an eight-point scale with lower values indicating a match based on a greater number of restrictions, i.e. matched on all identifiers. A lower value is therefore considered to be stronger evidence for a true positive match.

### CPRD generated linked data

Metadata generated during record linkage are provided to researchers to inform selection of denominator populations and study design. CPRD generates a source file containing patients from the CPRD cohort file supplied by NHS Digital which includes eligibility flags for each available dataset. Patients are considered eligible for a linkage if they have the required variables for the linkage and the patient has not opted-out. For example, patient primary care records matched to deprivation data by postcode are flagged as ineligible for this linkage if they do not have a valid postcode in the cohort file. Eligibility varies, with some patients eligible for all or some linkages and others not eligible for any linkages. Data pertaining to coverage start and end dates for each linked dataset are also provided. Collectively, these metadata allow users to accurately identify patients eligible for linkages of interest and relevant denominator populations. To ensure data security, data files are encoded with an additional layer of pseudonymisation prior to release to researchers.

CPRD provides standard and non-standard linked datasets based on match rank metadata generated during linkage. Standard datasets are designed to minimise the probability of false matches and include all patients with a match rank between 1 and 5 and a single-to-single primary care to external dataset match, i.e. one primary care record matched to one record in an external dataset. Many to single matches are also included when multiple primary care records are matched to a single record in the external dataset, i.e. when patients have moved or been registered at more than one practice contributing data to CPRD. As match ranks 1–5 require agreement on NHS number, linkage eligibility for data sources linked to CPRD standard datasets using the stepwise algorithm is set to zero if a valid NHS number was not transferred to NHS Digital. Table 2 shows the proportion of CPRD patients linked to patients with secondary care data from the Hospital Episode Statistics (HES) dataset at each step of the linkage algorithm for the three most recent linkage sets. The majority of patients (~ 96%) are matched on steps 1 and 2, with less than 4% matched on ranks 6–8, and this was consistent between linkage sets.

**Table 2:** Proportion of patients matched in CPRD GOLD-HES linkage at each match rank for the three most recent linkage sets

	Linkage set version 14 June 2017	Linkage set version 15 December 2017	Linkage set version 16 June 2018
Patients in CPRD GOLD cohort	10,425,601	10,494,935	10,553,586
Patients eligible to be linked to HES data in CPRD standard linked dataset	8,328,954	8,391,529	8,444,946
Patients matched to HES on match rank 1	5,098,291 (67.19%)	5,186,589 (67.50%)	5,241,901 (67.59%)
Patients matched to HES on match rank 2	2,204,352 (29.05%)	2,211,157 (28.78%)	2,227,150 (28.72%)
Patients matched to HES on match rank 3	13,316 (0.18%)	13,318 (0.17%)	13,344 (0.17%)
Patients matched to HES on match rank 4	17,241 (0.23%)	17,385 (0.23%)	17,528 (0.23%)
Patients matched to HES on match rank 5	3678 (0.05%)	3600 (0.05%)	3567 (0.05%)
Patients matched to HES on match rank 6	232,331 (3.06%)	232,287 (3.02%)	232,007 (2.99%)
Patients matched to HES on match rank 7	13,730 (0.18%)	13,948 (0.18%)	13,992 (0.18%)
Patients matched to HES on match rank 8	5483 (0.07%)	5431 (0.07%)	5396 (0.07%)

As of June 2018, the latest set of linkage data, referred to as set 16, is available for both CPRD GOLD, based on the Vision software system, and CPRD Aurum, based on EMIS software. This table is based on the CPRD GOLD data

Non-standard datasets containing patients with a match rank between 6 and 8 can be provided on request with a source file in which linkage eligibility flags do not depend on NHS number. One-to-many matches, that is, primary care records that match to multiple external records, can also be provided separately, but may represent linkage errors.

Linked datasets may be formatted as necessary prior to release, including transformation into a normalised data structure, e.g. wide to long, and the creation of derived variables (e.g. most commonly recorded ethnicity for a patient from all HES records), to facilitate relevant analyses. CPRD provides documentation and data dictionaries for the source file and all linked data sources. The match rank variable is included in each dataset and its distribution described in the documentation.

It is important to note that external datasets may undergo a prior, additional linkage process before being linked to CPRD primary care data. For example, records from individual hospital visits by the same patient are matched to create the HES datasets, which are subsequently linked to CPRD primary care data. False and missed matches in the HES linkage algorithm have been reported at 0.2% and 4.1% respectively, for paediatric intensive care records [22]. Any errors in the original HES linkage are likely to be compounded during linkage to CPRD primary care data. Similarly, HES data are linked to the Diagnostic Imaging Dataset to create the HES DIDs dataset, which is subsequently linked to CPRD primary care data. Errors in the HES DIDs linkage process will be carried forward to the linkage with CPRD data.

## Discussion

Record linkage is a powerful and established tool to improve the accuracy and completeness of patient information used for public health research purposes [3–5, 23]. CPRD is a major provider of routinely linked primary care and other patient data in England. Similar linkage projects include the US SEER-Medicare database [7] combining cancer registry data with national social insurance claims data and the Canadian Institute for Clinical Evaluative Sciences [8] linking administrative health data to population and census data, registries and survey data. Within Europe, the PHARMO record linkage system [9] in the Netherlands and Statistics Denmark [10] link various national patient data on prescriptions, hospital visits, death certificates and registries. Within the UK, the closest available comparators to CPRD are The Health Improvement Network (THIN) database [12], and the Secure Anonymised Information Linkage (SAIL) Databank in Wales [11, 24]. SAIL provides a range of routinely linked Welsh data, including primary care data, births, and deaths, hospitalisation, and demographic data. SAIL uses a combination of deterministic and probabilistic matching to link datasets based first on matching NHS numbers, then on deterministic matching of first name, surname, date of birth, sex and postcode with unmatched records being subjected to probabilistic matching. Using the first two steps of this linkage, SAIL is able to link 96.6% of records between primary and secondary care records, a very similar figure to the linkage obtained by the CPRD primary care data-HES linkage (Table 2).

Currently, greater than two-thirds of CPRD data access protocols request primary care data linked to other health-related datasets. Analyses of conditions or events with management in both primary and secondary care may benefit from using linked data [13, 17, 25]. Hospitalisation events in particular may be better recorded in secondary versus primary care [17]. For example, Herrett et al. [25] showed a significant improvement in the identification of myocardial infarction using linked primary care, HES, Office for National Statistics (ONS) and data from Myocardial Ischaemia National Audit Project (MINAP), with single sources underestimating rates by up to 50%. Similarly, Millet et al. found that the identification of community-acquired pneumonia using primary care or HES data differed by up to 83% between 1997 and 2010 due to a change in the recording of events over time [13]. Primary care data linked to other health-related datasets therefore has the potential to expand the scope of research and to improve the validity of study outcomes.

An understanding of data linkage methodology is essential for robust analysis and interpretation of linked data. In particular, the choice between multiple datasets can impact on the potential for systematic bias. Gallagher et al. [26] recently demonstrated a secondary care mortality rate due to venous thromboembolism almost double that recorded primary care, suggesting that data from different care settings may represent distinct populations and should be taken into account when evaluating event rates. Mortality rates due to venous thromboembolism also differed substantially when the coverage period of the linked data sources or linkage eligibility were not taken into account. Restricting linked data to participating practices, but not by using individual patient eligibility flags, led to a lower mortality rate, as did analysing a dataset including all primary care, HES and ONS data irrespective of coverage period. In this case, the lower mortality rate is likely due to missed ONS mortality records from patients ineligible for linkage, or the linkage coverage period of all datasets not being sufficient to cover the full study period. The finding highlights the importance of considering these factors in study design and interpretation of study findings.

Both erroneous, missing or incomplete data records, and the methodology used for record linkage, have the potential to introduce misclassification into research studies. Misclassification leads to bias if linkage accuracy differs between comparator groups. With both deterministic and probabilistic strategies, decisions made by data scientists or researchers affect the sensitivity and specificity of the approach. Deterministic linkages may be most appropriate when unique identifiers are common between datasets, the percentage of missing values is low and there is a clear hierarchy of identifiers; these cases will have a very high specificity [27]. Probabilistic strategies aim to remove subjectivity in rule setting when available matching variables are not unique, may be incomplete or missing and are likely to contain errors [18, 19].

The approach to linkage described here uses a stepwise deterministic method with a combination of unique and partial identifiers including, importantly, NHS number in the five most restrictive steps. The NHS number is a unique identifier and pseudonym which remains the same throughout an individual’s lifetime, and has previously been shown to be valid and complete for greater than 94% of HES records and 99.8% of primary care records in England [28]. Using NHS number in combination with other partial identifiers arguably reduces the potential for missed and/or false matches, both of which have been shown to introduce potential biases [22, 29–33]. Whilst this approach can be applied to data sources that do not record NHS number, including those outside the health domain, resulting linkages may, if not using an identifier of similar properties to the NHS number, be deemed to have a lower match quality.

This approach generates meaningful metadata that can be used to inform study design and interpretation of subsequent analyses. CPRD provides documentation for the source files and for each linked dataset. The documentation is updated regularly and includes recommendations for defining a linked patient cohort. For example, to identify patients in CPRD primary care data with overlapping follow-up in HES data, investigators should define the start and end of follow-up using the start and end of the HES coverage period, primary care practice and patient registration dates. Patients with no follow-up time and patients who are not in the linkage source file or marked ineligible for HES linkage should be excluded. This prevents misclassification of patients where the event occurred outside of the linked data coverage period or the necessary patient identifiers were not available for linkage; these patients would be classified as unexposed in cohort studies or controls in case control studies irrespective of whether the event occurred. Metadata provided on the match rank can be used to inform sensitivity analyses and the interpretation of findings in the context of possible misclassification or selection bias.

The approach to linkage adopted by CPRD and NHS Digital has resulted in a high proportion of research quality patients who are deemed acceptable for use in observational studies. In 2018, 10.6 million (M) patients from 411 English general practices participated in record linkage and constituted the CPRD GOLD source file generated in June 2018. 9.1M (86%) of these patients were of research quality, of which 8.0M (88%) had a valid NHS number and were eligible for linkage in the CPRD standard linked dataset release. The majority of patients who are not flagged as research quality by CPRD have a temporary registration in the practice and would not be suitable for inclusion in research studies.

Previous research has demonstrated the representativeness of the subset of patients eligible for linkage, in terms of age, gender and geography [34]. Whilst outside the scope of this work, further comparisons of indicators of general health and overall health service use by linkage eligibility would be a valuable area of future research. In addition, an analysis of patient characteristics by match rank could potentially identify subgroups most likely to be associated with false matches. At present, the match rank can be used to explore reasons for inconsistent data, further research is required to establish whether matches with higher ranks are more valid, whether this varies over time or by subgroup.

Greater transparency with respect to methodology has been put forward as best practice for linkage, in order to enable high quality research [35, 36]. Governance procedures commonly specify that linkage is carried out by a trusted third party, and data custodians are not permitted to release identifiable details to CPRD or to researchers to protect patient anonymity. Frequently, this means that data users lack information on linkage methodology to inform research decisions. In this paper, CPRD and NHS Digital have endeavoured to report the approach to linking primary care data to external patient level datasets in line with the recent GUILD publication and reporting guidelines for observational research [3, 20]. A limitation of this paper is that it is a descriptive report of the current approach and does not include a validation study or comparison with alternative deterministic or probabilistic methodologies.

Ongoing assessment and input from data users will further improve the strategies used to link CPRD primary care data to external datasets. CPRD encourages user feedback, validation studies and collaborative projects to further strengthen observational research conducted using CPRD linked data.
